# Transcriptomic analysis of DENV-2-infected human dermal fibroblasts identified potential mechanisms that suppressed ZIKV replication during sequential coinfection

**DOI:** 10.1186/s12985-025-02769-9

**Published:** 2025-05-22

**Authors:** Chernkhwan Kaofai, Tuksin Jearanaiwitayakul, Khwankhao Saisingha, Jitra Limthongkul, Promsin Masrinoul, Sukathida Ubol

**Affiliations:** 1 Department of Microbiology, Faculty of Science, Mahidol University, Bangkok, Thailand; 2 Department of Clinical Pathology, Faculty of Medicine Vajira Hospital, Navamindradhiraj University, Bangkok, Thailand; 3 Center for Vaccine Development, Institute of Molecular Biosciences, Mahidol University, Nakhon Pathom, Thailand

**Keywords:** Dengue virus, Zika virus, Coinfection, Human dermal fibroblasts, Transcriptomic profiling, Superinfection exclusion

## Abstract

Dengue virus (DENV) and Zika virus (ZIKV) are closely related flaviviruses which are transmitted by the same species of mosquitoes. Due to overlapping geographic distributions and transmission vectors, cases of DENV-ZIKV coinfection have been reported. However, the impact of coinfection on disease outcomes remains unclear. In this study, an in vitro model of DENV-ZIKV coinfection was developed using the primary human dermal fibroblasts (HDFs). The interaction between DENV-2 and ZIKV during sequential coinfection revealed that prior DENV-2 infection significantly suppressed ZIKV RNA accumulation in the culture supernatant. Transcriptomic profile in response to DENV-2 infection suggested three hypothetical pathways that potentially interfere with ZIKV replication. The first mechanism is prior DENV infection drove HDFs into an antiviral state through upregulation of genes involving innate immune response pathways, including PRR signaling, type I and type II IFN signaling, ISG activity, and cytokine/chemokine activity. This state significantly enhanced resistance to subsequent ZIKV infection in both infected cells and uninfected neighboring cells. The second potential pathway is inhibition of viral entry. This was supported by DENV-2-infected HDFs significantly suppressed expression of ZIKV receptor and reduced expression of genes involving in clathrin-mediated endocytosis. This can interfere with entry of ZIKV into host cells. The last possible mechanism is driving cells into cell cycle arrest, as DENV-2 infection downregulated genes related to cell cycle progression, which may hinder ZIKV replication. These findings partly unfold the interplay between DENV and ZIKV at the entry site which may explain the disease outcome of DENV-ZIKV coinfection.

**Supplementary Information:**

The online version contains supplementary material available at 10.1186/s12985-025-02769-9.

## Background

Viral coinfection has emerged as a critical public health challenge, as these interactions may affect viral replication, immune responses, and disease outcomes. Thus, understanding the interplay between these viruses in the target host is significance for effective managing strategies. DENV and ZIKV are closely related flaviviruses transmitted by the same species of mosquito vector, *Aedes aegypti*, which can simultaneously be infected with various arboviruses and subsequently transmit them in a single bite [1]. Of note, during the major ZIKV outbreaks, there were reports of DENV-ZIKV coinfected patients in several countries, including Thailand, Brazil, and Nigeria [2–4]. However, current clinical data are insufficient to assess the disease severity of DENV-ZIKV coinfections in these individuals.

On the other hand, coinfection outcomes of other viruses have been reported. For example, an increasing number of cases concurrently infected with DENV and the emerging SARS-CoV-2 was reported [5] and regions with high DENV prevalence showed milder SARS-CoV-2 cases, possibly due to immunological cross-reactivity [6]. In addition, simultaneously infections of seasonal respiratory viruses have been observed in the host. Irrespective of the sequence of exposure, SARS-CoV-2 and influenza A virus exhibited mutual interference in their replication kinetics [7–10] but in mouse model this coinfection increased lung damage [11]. Moreover, coinfection between respiratory syncytial virus (RSV) and SARS-CoV-2 in the respiratory tract was reported to result in the inhibition of SARS-CoV-2 propagation while prior RSV infection did not reduce SARS-CoV-2 replication [8, 12].

In contrast to the human host in which the effects of simultaneous and sequential arboviruses coinfection remain inconclusive, several studies have reported arboviruses coinfection outcomes in *Aedes* mosquitoes. For example, sequential DENV-ZIKV coinfection in *Ae*. *aegypti* resulted in suppression of secondary ZIKV replication [13]. Conversely, primary infection with DENV or chikungunya virus (CHIKV) in *Ae. aegypti* enhanced ZIKV replication [14, 15]. In addition, prior DENV exposure enhanced CHIKV replication [16]. While DENV-ZIKV coinfection has been primarily studied in mosquitoes, the effects of DENV-2 and ZIKV interactions in human skin cells, which are the primary target cells following a mosquito bite, remain largely unexplored.

In this study, we investigated the interactions between DENV-2 and ZIKV during coinfection in HDFs, including both simultaneous and sequential coinfections. Sequential coinfection refers to a condition that HDF cells were primarily infected with DENV-2 for 24 h before being superinfected with ZIKV. In the present work, effects of DENV-2 on ZIKV infection, but not other way around, was focused. This is because Thailand is a hyperendemic area for DENV, with year-round circulation of all four serotypes, while ZIKV outbreaks occur sporadically. We found that primary DENV-2 infection induced ZIKV replication exclusion in HDFs. Transcriptomic analysis suggested that DENV-2 infected HDFs were driven into an antiviral state, which may be a major mechanism restricting ZIKV replication. Concurrently, DENV-2 infection downregulated genes associated with cell cycle regulation, decreased expression of viral receptors, and suppressed clathrin-mediated endocytosis. All of these events may participate in resistance against subsequent ZIKV infection.

## Methods

### Cell cultures and virus

Vero cells and C6/36 cells were cultured in MEM containing 10% FBS and 1% L-Glutamine. Human dermal fibroblasts (HDFs) were cultured in DMEM containing 10% FBS, 1% L-Glutamine, and 1% Penicillin-Streptomycin. DENV-2 (strain 16681) (GenBank accession no. U87411.1) and ZIKV (strain SV0010/15) (GenBank accession no. KX051562) were propagated in C6/36 cells and titrated using plaque assay in Vero cells.

### DENV-2 and ZIKV infection in HDFs culture

For DENV-2 or ZIKV monoinfection, HDFs were infected with DENV-2 at MOI of 5 or ZIKV at MOI of 10. For simultaneous coinfection, HDFs were simultaneously infected with DENV-2 at MOI of 5 and ZIKV at MOI of 10. For sequential coinfection, HDFs were primarily infected with DENV-2 at MOI of 5 for 24 h before being super-infected with ZIKV at MOI of 10. At 24, 48, and 72 h of infection, HDF culture supernatant was aliquoted and subjected to viral RNA copies determination using qRT-PCR and cytokine production using ELISA.

### Quantitative RT-PCR (qRT-PCR)

The qRT-PCR was used to quantify viral RNA copies and validate of gene expression level. To quantitate the copy numbers of DENV-2 and ZIKV genomes, total RNAs were extracted from the supernatant using TRIzol reagent (Invitrogen, USA) following the manufacturer’s protocol. Purified RNAs were reverse-transcribed into cDNA using AMV Reverse Transcriptase (Promega, USA) with 1X AMV reverse transcriptase buffer, 0.1 M DTT, 1 mM dNTP, 0.5 µg random hexamer primer, 8 U RNase inhibitor, and 8 U AMV reverse transcriptase. Reactions were incubated at 37 °C for 60 min, and the cDNA was stored at -20 °C. qPCR was performed using the 2X KAPA SYBR^®^ FAST qPCR kit master mix (KAPA Biosystems, USA) and 10 µM gene-specific primers on a Mic real-time PCR machine (Bio Molecular Systems, USA). The primer sets specific for DENV-2 and ZIKV were shown in Additional file 1. The thermal cycling conditions were 95 °C for 5 min, followed by 35 cycles of 95 °C for 20 s, 60 °C for 30 s, and 72 °C for 30 s. Viral RNA copies were quantified by comparing the sample’s threshold cycle (Ct) value with the Ct values of known concentrations (copies/µL) from a beta-actin RNA standard curve.

To validate the level of gene expression, total RNAs were extracted from infected cells using the PureLink™ RNA Mini Kit (Invitrogen, USA) according to the manufacturer’s protocol. Reverse transcription was performed using AMV Reverse Transcriptase with oligo dT primers under the same conditions as previously described. The reactions were performed at 42 °C for 60 min. qPCR was carried out using the 2X KAPA SYBR^®^ FAST qPCR kit master mix and 10 µM gene-specific primers (Additional file 1). Primers were designed using NCBI Primer-BLAST and obtained from published data. The thermal cycling conditions were 95 °C for 5 min, followed by 35 cycles of 95 °C for 20 s, 66 °C for 30 s, and 72 °C for 30 s. Human GAPDH was used as a reference gene. Relative fold changes in gene expression were calculated using the 2^−ΔΔCt^ method, compared to the expression in the mock-infected cells.

### Quantification of infected cells by flow cytometry

The infected cells were fixed and permeabilized using Cytofix/Cytoperm™ solution kit (BD biosciences, USA) and processed for intracellular staining using mouse anti-Flavivirus envelope protein antibody (4G2) and Alexa Fluor 488-conjugated goat anti-mouse IgG (Invitrogen, USA). The mean fluorescence intensity (MFI) and frequency of Alexa Fluor 488 positive cells were assessed by CytoFLEX Flow Cytometer (Beckman Coulter, USA).

### Transcriptome profiling of DENV-2 infected HDFs using RNA sequencing

The infected cells were subjected to total RNA extraction using the PureLink™ RNA Mini Kit (Invitrogen, USA) following the manufacturer’s instruction. RNA sequencing using TruSeq was conducted by Humanizing Genomics Macrogen, Inc., The Republic of Korea. Briefly, mRNAs were captured through poly-A selection and reverse transcribed into cDNAs for paired-end RNA sequencing. Quality control of the raw sequenced reads was performed using FastQC, and low-quality reads were filtered out with Trimmomatic. The trimmed reads were then mapped to the reference genome (GRCh37) using HISAT2 via Bowtie2. Lastly, StringTie was used to quantify the raw read counts.

Data landscape visualization was performed using the integrated differential expression and pathway analysis (iDEP) web platform. Differentially expressed genes (DEGs) were identified using DESeq2, with significant DEGs selected based on an FDR-adjusted *p*-value ≤ 0.05 and an absolute log2FoldChange ≥ 1. Volcano plots were generated using the EnhancedVolcano package in R. Gene Ontology (GO) analysis was conducted for functional and pathway enrichment using the clusterProfiler package in R. The protein-protein interaction (PPI) network was generated using the STRING database, and module analysis was performed with Molecular Complex Detection (MCODE) to identify densely interconnected regions (modules) within the network.

### Validation of biological activities of DENV-2-infected HDFs supernatant

DENV-2-infected HDF supernatant was subjected to UVA radiation (254 nm, 576 µW/cm² at a 15 cm distance, 1,555.2 mJ/cm²) for 30 min to inactivate the virus. Inactivation efficiency was validated using plaque assay in Vero cells. To determine the inhibitory activity in the supernatant, HDF cultures were treated with UV-inactivated supernatant from DENV-2-infected cells for 24 h. These treated HDFs were then infected with ZIKV at MOI of 5 for 24 h. ZIKV infection was quantified by flow cytometry.

### Statistical analysis

Data were analyzed using GraphPad Prism 9.0 (GraphPad Software) with Student’s *t*-test. Results were presented as mean ± standard error of the mean (SEM) or standard deviation (SD). All experiments were performed in three independent experiments, and differences were considered statistically significant at *p*-value < 0.05.

## Results

### DENV-2 infection significantly suppresses subsequent ZIKV infection

Since DENV and ZIKV cocirculate in the same endemic regions, therefore, coinfection of these viruses can occur in both mosquitoes and individuals [3, 13]. Given this context, it is interesting to investigate the interplay between these viruses in the frontline target cells, HDFs, in the human host. To assess the permissiveness of HDFs to DENV-2 and ZIKV infections, HDFs were infected with various MOIs of each virus. Viral RNA production in the culture supernatant was quantified using qRT-PCR. The results revealed that infection with DENV-2 or ZIKV at an MOI of 5 and 10, respectively, yielded non-different levels of replication efficiency at 24 h and onwards (Additional file 2). Therefore, the following experimentations were performed using DENV-2 and ZIKV at the MOI of 5 and 10, respectively.

**Fig. 1:**
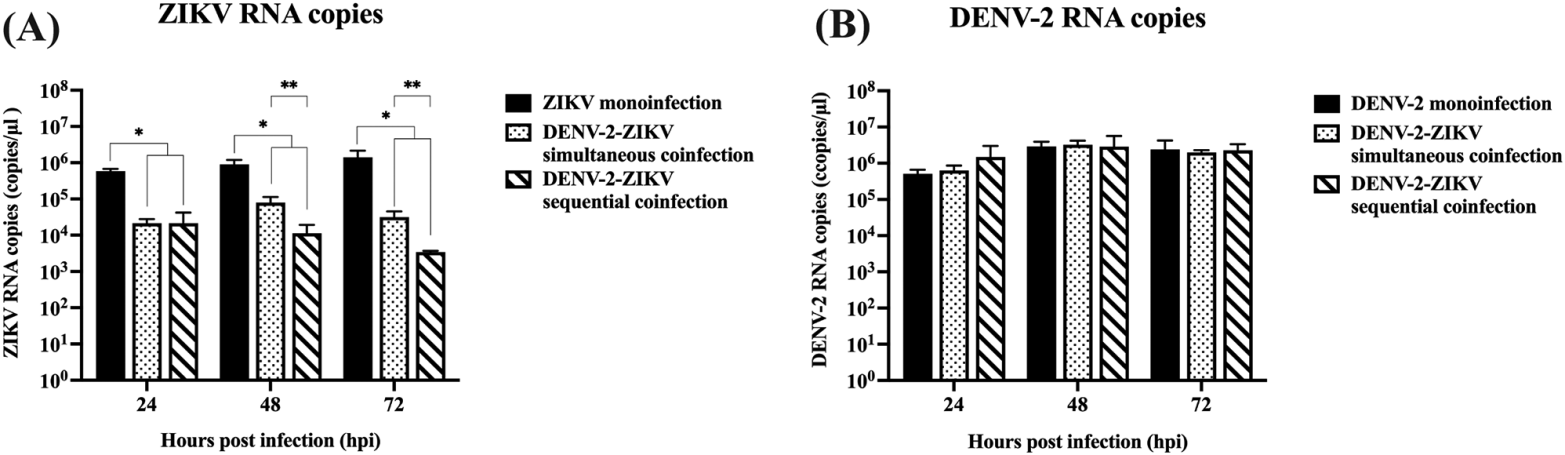
Viral RNA accumulation during DENV-2-ZIKV coinfection in HDF cells. HDF cells were coinfected with DENV-2 (MOI of 5) and ZIKV (MOI of 10). The RNA levels of DENV-2 and ZIKV were measured by qRT-PCR at 24, 48, and 72 hpi. Compared to the monoinfected cells, ZIKV RNA levels were significantly reduced (**A**), while DENV-2 RNA levels in coinfected cells were not significantly different from those in cells infected with DENV-2 alone (**B**). Data were presented as mean ± SEM from three independent experiments and analyzed using Student’s *t*-test (*p* < 0.05). * indicated significant differences between monoinfection and simultaneous/sequential coinfection, while ** indicated significant differences between simultaneous and sequential coinfections

To investigate interactions between DENV-2 and ZIKV in HDFs, cells were subjected to simultaneous or sequential coinfections as described in Methods section. Viral RNA levels in the supernatant were monitored using qRT-PCR. The findings revealed that ZIKV replication in simultaneous and sequential coinfections was significantly suppressed compared to ZIKV monoinfection (Fig. 1A). Notably, ZIKV RNA copies in sequential coinfection were significantly lower than that of simultaneous coinfection at both 48 and 72 hpi (Fig. 1A). The suppressive effect was more pronounced in condition that HDF cells were primarily infected with DENV-2. Surprisingly, neither simultaneous- nor sequential-coinfection had suppressive effect on DENV-2 RNA accumulation detected in the culture supernatant (Fig. 1B).

### Transcriptome profiling in HDFs in response to DENV-2 infection

As shown in Fig. 1A, DENV-2-infected HDF cells exerted the suppressive effect on the replication of ZIKV during both types of coinfections. Therefore, transcriptome profiling of DENV-2-infected HDFs was monitored to identify host genes that may potentially be involved in inhibiting ZIKV replication. HDFs were infected with DENV-2. The numbers of infected cells were monitored and found to peak at 16 and 24 hpi (64.26% and 65.84% of infection, respectively) (Additional file 3), making these the optimal time points for transcriptome sequencing.

**Fig. 2:**
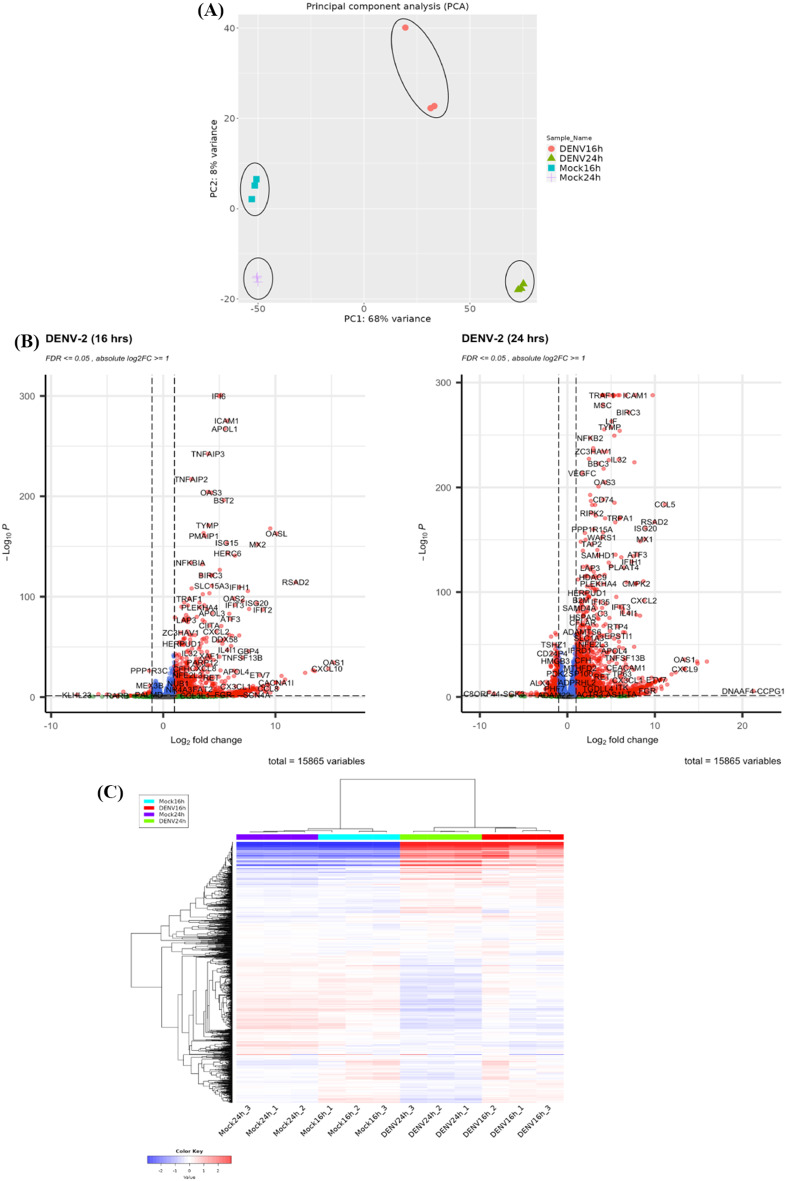
Global transcriptome and DEGs of HDFs in response to DENV-2 infection. (**A**) PCA of HDFs in response to DENV-2 or mock infection. (**B** and **C**) To identify DEGs, data were analyzed using DESeq2 in the iDEP web application by comparing DENV-2-infected samples with mock controls at 16 and 24 hpi. Significant DEGs were selected with an FDR-adjusted *p*-value ≤ 0.05 and │log2FoldChange│≥ 1. (**B**) The DEGs were expressed as Volcano plots. Red dots represented significant DEGs, gray dots represented non-DEGs, green dots represented genes with │log2FoldChange│≥ 1 but *p*-value > 0.05, and blue dots represented genes with *p*-value ≤ 0.05 but │log2FoldChange│< 1. (**C**) The identified DEGs were revealed as heatmap between DENV-2- or mock-infected HDFs

To assess global changes in gene expression, DENV-2-infected HDFs cultures were harvested at 16 and 24 hpi. The total RNAs were extracted, purified and subjected to transcriptome sequencing. Quantified transcript reads were transformed and normalized to reduce mean-dependent variance (Additional file 4). PCA revealed distinct clustering between mock and DENV-2-infected samples along the first principal component (PC1), which accounted for 68% of the variance, underscoring significant transcriptomic alterations in response to DENV-2 infection. In addition, no significant variation observed among the three replicates within each infection condition (Fig. 2A). To identify differentially expressed genes (DEGs), the normalized data was further analyzed using DESeq2 in the iDEP web application. The results were presented using Volcano plots and heatmap. Volcano plots indicated that transcriptomic changes were more pronounced at 24 hpi compared to 16 hpi (Fig. 2B). This corresponded to the higher number of upregulated and downregulated genes at 24 hpi, with 1828 upregulated and 397 downregulated genes. In contrast, the number of upregulated and downregulated genes at 16 hpi were 953 and 51, respectively. Heatmap showed distinct transcriptional profiles between DENV-2-infected and mock-infected cells, with marked gene expression changes particularly evident at 24 hpi (Fig. 2C). This reflected a time-dependent increase in gene expression changes in HDFs in response to DENV-2 infection.

### Functional enrichment of the transcriptome profile in DENV-2-infected HDFs

**Fig. 3:**
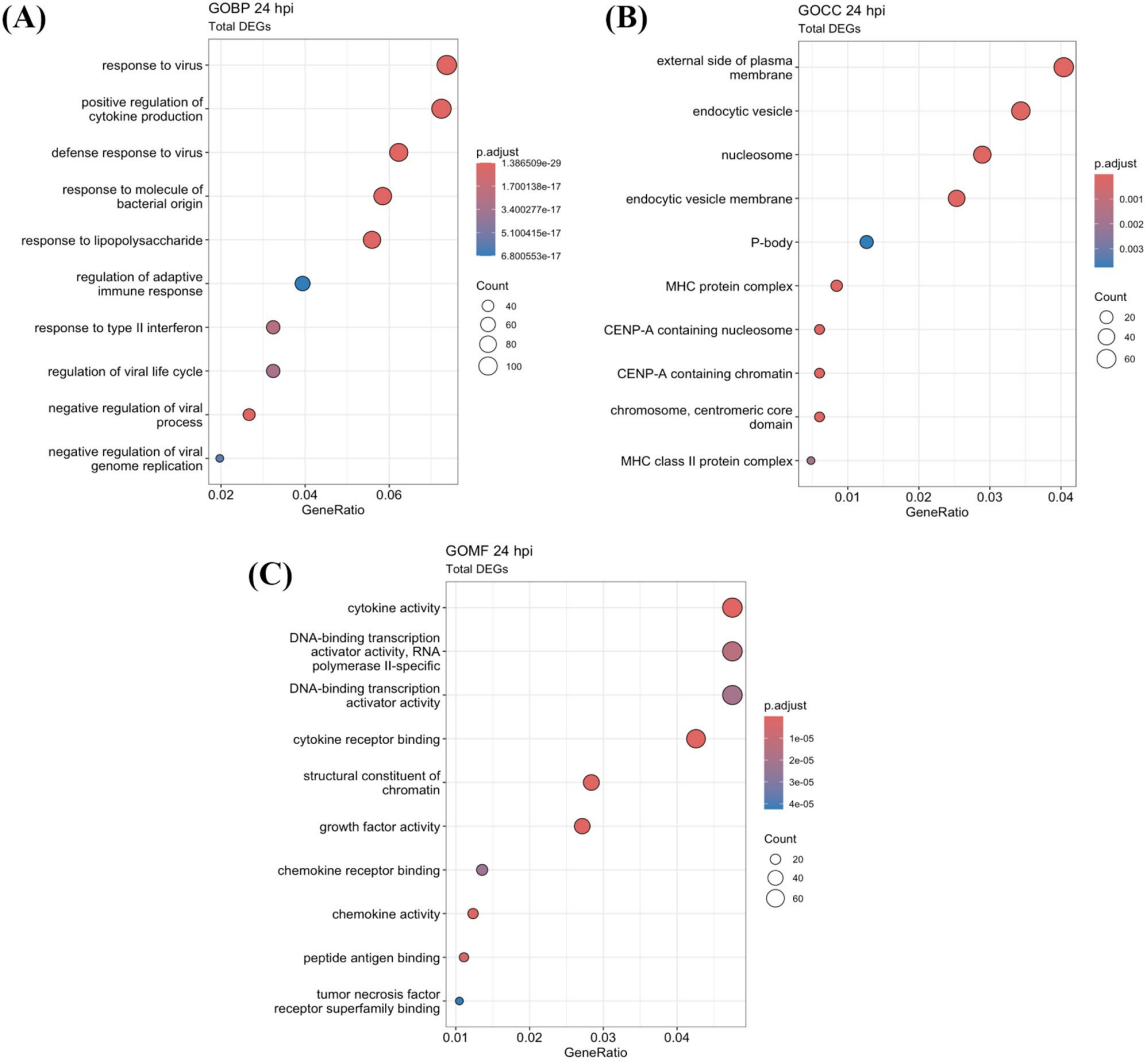
Functional enrichment analysis of total DEGs in DENV-2-infected HDFs at 24 hpi. Total DEGs at 24 hpi, encompassing both upregulated and downregulated DEGs, was subjected to Gene Ontology (GO) enrichment analysis. The top 10 enriched pathways were displayed for biological processes (**A**), cellular components (**B**), and molecular functions (**C**). Colored dots represented distinct significant enrichment (*p* ≤ 0.05). Dot size represented the number of genes

To explore host response pathways in DENV-2-infected HDFs that have potential to inhibit ZIKV replication, functional enrichment analysis of total DEGs was conducted using the Gene Ontology (GO) database. The GO terms identified in infected cells at 24 hpi were also found in the pathways of the total DEGs from infected cells at 16 hpi, but the 24 hpi samples exhibited a significantly higher number of associated genes. This suggested that an early host response occurred at 16 hpi, with an intensified response at 24 hpi (Fig. 3 and Additional file 5). Key biological processes were primarily associated with the activation of the innate immune response and the control of viral replication, including response to virus, positive regulation of cytokine production, response to type II interferon, and regulation of the viral life cycle. These processes began at 16 hpi and were sustained over 24 hpi (Fig. 3A and Additional file 5). Cellular components mainly involved in activities that took place at the external side of the plasma membrane, endocytic vesicles, and nucleosomes (Fig. 3B and Additional file 5). Molecular functions highlighted cytokine activity, structural constituent of chromatin, growth factor activity, and chemokine activity (Fig. 3C and Additional file 5). Only infected cells at 24 hpi enriched DNA-binding transcription activator activity (Fig. 3C), while only infected cells at 16 hpi were related to protein heterodimerization (Additional file 5). How these pathways contribute to inhibiting ZIKV replication is of interest.

### Clusters of upregulated DEGs that potentially participate in viral interference

**Fig. 4:**
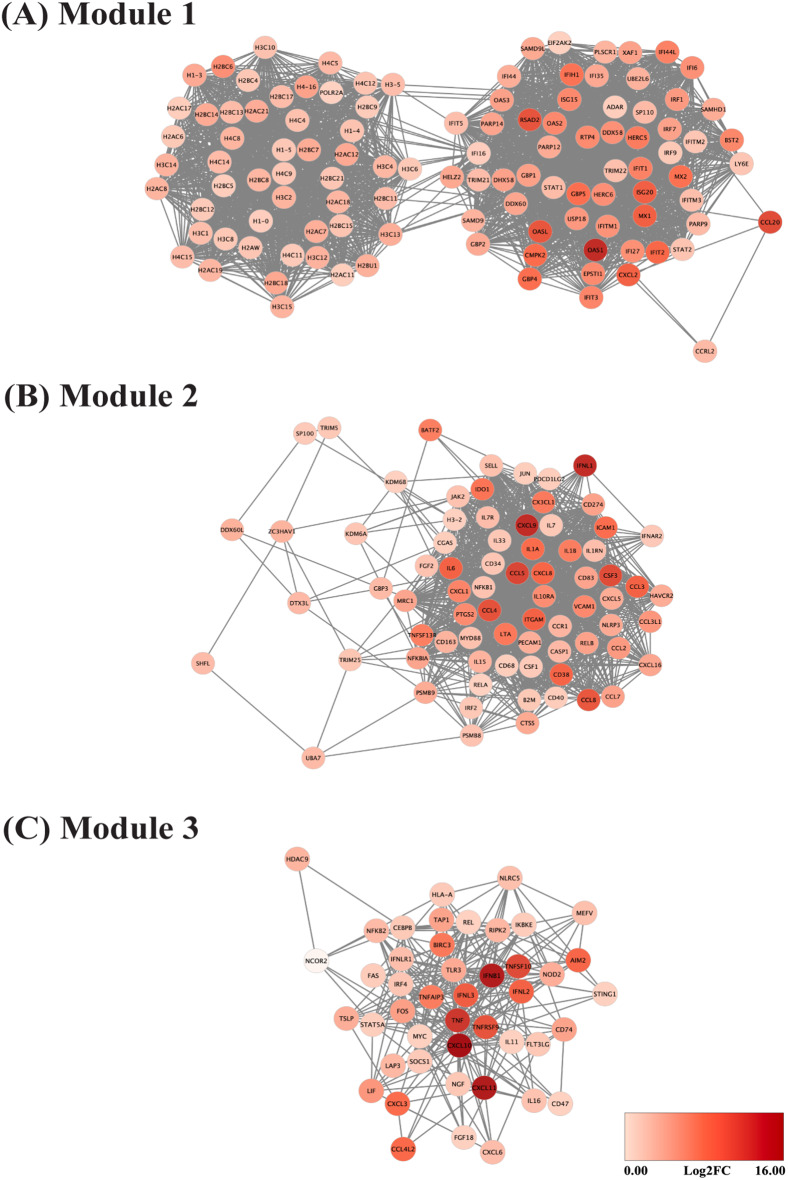
Clusters of upregulated DEGs potentially implicated in viral interference. The significantly upregulated DEGs at 24 hpi, selected based on an FDR-adjusted *p*-value ≤ 0.05 and log2FoldChange ≥ 1, were input into the STRING database to generate PPI networks and visualized in Cytoscape. Highly interconnected clusters were identified with MCODE, including module 1 (**A**), module 2 (**B**), and module 3 (**C**)

To identify clusters of upregulated DEGs that may involve in superinfection exclusion of ZIKV, PPI networks were constructed using the STRING database and visualized in Cytoscape. The three most significant modules, characterized by highly interconnected regions, were identified using the MCODE algorithm. The first gene cluster that may disrupt ZIKV replication were transcripts involving in the innate immune response and nucleosome, as shown in the top three significant modules among the upregulated DEGs at 24 hpi (Fig. 4A-C). Interestingly, module 1 revealed an association between genes from two main functional groups: nucleosome and innate immune response (Fig. 4A). Key genes in the nucleosome pathways encoded histone proteins. Cluster of innate immune response was further analyzed using the functional enrichment, revealing significant enrichment for pattern recognition receptor (PRR) signaling pathways (NOD-like, RIG-I-like, C-type lectin, and cytosolic DNA-sensing pathways) and interferon signaling (interferon alpha/beta, interferon gamma, and antiviral mechanisms mediated by ISGs) (Table 1). Module 2 was associated with cytokine and chemokine activities (Fig. 4B), enriched for inflammatory response, CCR and CXCR chemokine receptor binding, and regulation of apoptotic processes (Table 1). Module 3 was primarily associated with cytokine responses (Fig. 4C), with subgroups involved in interleukin signaling and Toll-like receptor cascades (Table 1). Similarly, three modules identified at 16 hpi were mainly related to innate immune response (Module 1), cytokine and chemokine activities (Module 2), and nucleosome and defense response to virus (Module 3), respectively (Additional file 6).

**Table 1:** Functional enrichment of subclusters within each module of upregulated DEGs

Module	Pathway	FDR value	Genes
Module 1 (Fig. 4A)	hsa04621: NOD-like receptor signaling pathway	3.11E-11	*OAS3*, *STAT2*, *OAS2*, *STAT1*, *GBP5*, *GBP4*, *GBP2*, *GBP1*, *IRF9*, *IRF7*, *OAS1*, *CXCL2*
	hsa04622: RIG-I-like receptor signaling pathway	1.4E-04	*DHX58*, *DDX58*, *IRF7*, *ISG15*, *IFIH1*
	hsa04625: C-type lectin receptor signaling pathway	0.0095	*IRF1*, *STAT2*, *STAT1*, *IRF9*
	hsa04623: Cytosolic DNA-sensing pathway	0.0299	*ADAR*, *DDX58*, *IRF7*
	HSA-909,733: Interferon alpha/beta signaling	3.61E-51	*USP18*, *OAS3*, *IRF1*, *BST2*, *OASL*, *ISG20*, *STAT2*, *IFITM1*, *MX2*, *OAS2*, *IFI6*, *STAT1*, XA*F1*, *ADAR*, *GBP2*, *IFIT5*, *IFIT1*, *IFIT3*, *RSAD2*, *IRF9*, *IRF7*, *MX1*, *IFITM3*, *OAS1*, *IFI35*, *IFI27*, *IFITM2*, *IFIT2*, *SAMHD1*, *ISG15*
	HSA-877,300: Interferon gamma signaling	3.53E-17	*OAS3*, *IRF1*, *TRIM21*, *OASL*, *OAS2*, *STAT1*, *GBP5*, *GBP4*, *GBP2*, *GBP1*, *TRIM22*, *IRF9*, *IRF7*, *OAS1*
	HSA-1,169,410: Antiviral mechanism by IFN-stimulated genes	1.3E-17	*USP18*, *OAS3*, *EIF2AK2*, *OASL*, *HERC5*, *UBE2L6*, *MX2*, *OAS2*, *STAT1*, *IFIT1*, *DDX58*, *MX1*, *OAS1*, *ISG15*
Module 2 (Fig. 4B)	GO:0042981: Regulation of apoptotic process	2.79E-07	*CX3CL1*, *CCL2*, *CD38*, *NFKB1*, *IL1A*, *IL1B*, *IL7*, *ICAM1*, *IL7R*, *SP100*, *PTGS2*, *JUN*, *CD40*, *CD274*, *JAK2*, *RELA*, *IL6*, *LT*A, *IDO1*, *CASP1*, *CCL5*, *CCL3*, *ITGAM*
	GO:0006954: Inflammatory response	2.31E-32	*CX3CL1*, *RELB*, *CCL2*, *NFKB1*, *CD68*, *KDM6B*, *IL1RN*, *IL1A*, *IL1B*, *VCAM1*, *CXCL5*, *CCR1*, *IL15*, *CXCL8*, *HAVCR2*, *CSF1*, *NLRP3*, *CD163*, *CXCL9*, *PTGS2*, *JUN*, *CD40*, *CCL7*, *IL33*, *JAK2*, *CCL8*, *CXCL1*, *RELA*, *IL6*, *IDO1*, *CCL5*, *CCL3*, *CCL3L1*, *CCL4*, *ITGAM*, *MYD88*
	GO:0048020: CCR chemokine receptor binding	1.38E-08	*CX3CL1*, *CCL2*, *CCL7*, *CCL8*, *CCL5*, *CCL3*, *CCL3L1*, *CCL4*
	GO:0045236: CXCR chemokine receptor binding	7.7E-06	*CX3CL1*, *CXCL5*, *CXCL8*, *CXCL9*, *CXCL1*
Module 3 (Fig. 4C)	HSA-449,147: Signaling by Interleukins	3.27E-15	*RIPK2*, *LIF*, *NLRC5*, *IL11*, *NOD2*, *IL16*, *CXCL10*, *FOS*, *IFNLR1*, *IFNL2*, *TSLP*, *STAT5A*, *NFKB2*, *IRF4*, *TNF*, *MYC*, *IFNL3*, *SOCS1*
	HSA-168,898: Toll-like Receptor Cascades	5.43E-08	*RIPK2*, *NLRC5*, *BIRC3*, *TLR3*, *NOD2*, *FOS*, *NFKB2*, *IKBKE*, *SOCS1*

### Clusters of downregulated DEGs that may be involved in viral interference

**Fig. 5:**
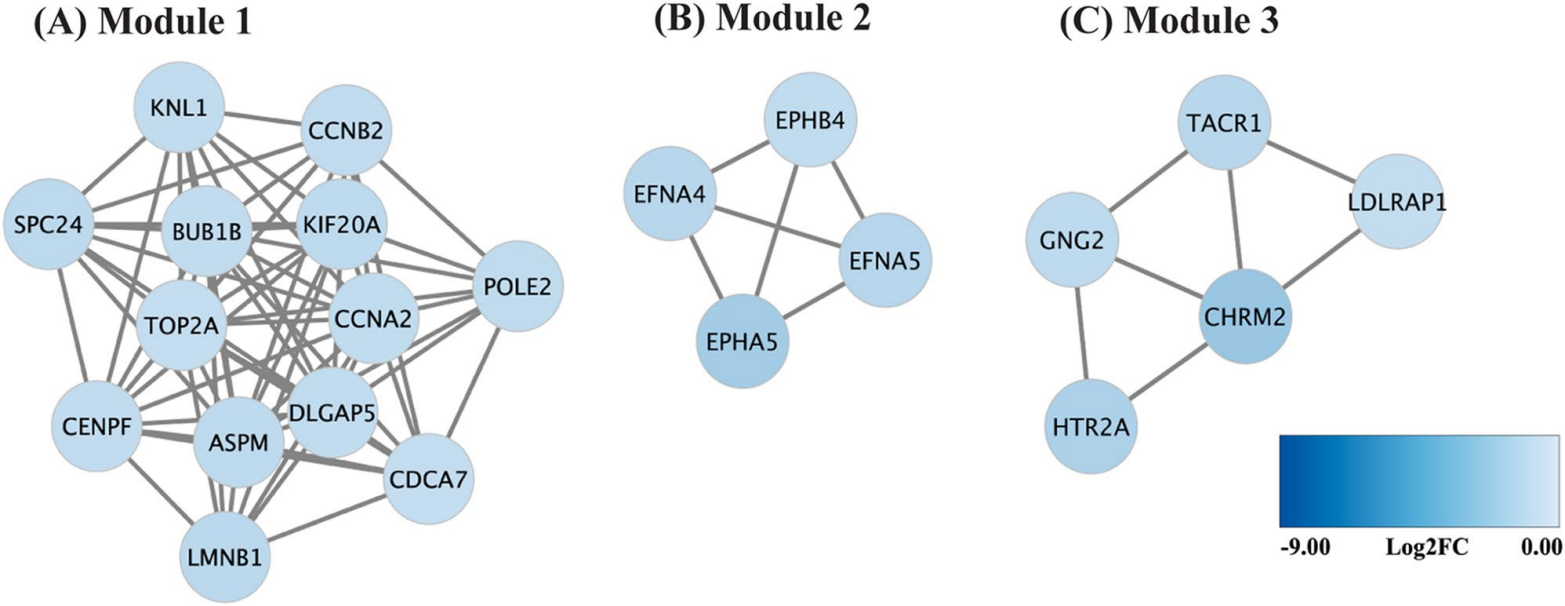
Clusters of downregulated DEGs potentially involved in viral interference. The significantly downregulated DEGs at 24 hpi, selected based on an FDR-adjusted *p*-value ≤ 0.05 and log2FoldChange ≤ -1, were input into the STRING database to generate PPI networks and visualized in Cytoscape. Highly interconnected clusters were identified with MCODE, including module 1 (**A**), module 2 (**B**), and module 3 (**C**)

To identify additional potential gene clusters involved in viral interference, downregulated DEGs were subjected to generate PPI networks and module analysis. Unfortunately, PPI networks and module analysis of downregulated genes at 16 hpi could not be performed due to the low number of downregulated genes. Thus, only downregulated DEGs at 24 hpi was analyzed. The potential gene clusters that may interfere with ZIKV replication was identified such as cell cycle processes, as shown in Module 1. Key genes in these processes included *TOP2A*, *CENPF*, *BUB1B*, *CCNA2*, and *CCNB2* (Fig. 5A). The additional clusters were groups of genes that affect the viral entry step, involving ephrin receptor signaling pathways (Module 2, Fig. 5B) and cargo recognition for clathrin-mediated endocytosis (Module 3, Fig. 5C). Importantly, *TYRO3* gene (log2FC = -0.62), receptor for ZIKV entry in human skin cells, was found to be downregulated in this study.

### Validation of transcriptomic profile

**Fig. 6:**
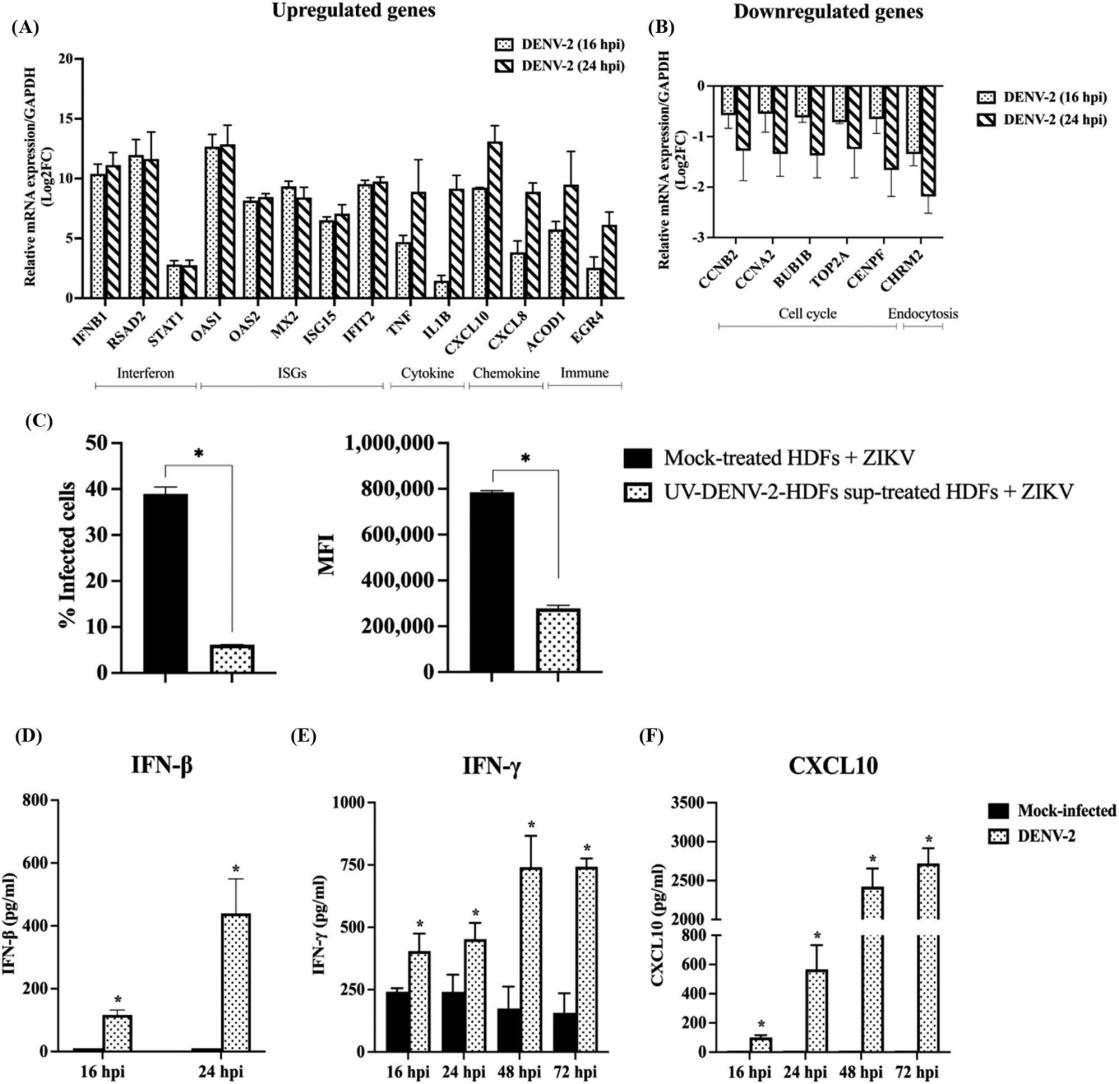
Validation of gene expression levels and biological activities in DENV-2-infected HDFs. Validation of gene expression levels using qRT-PCR. Total RNAs were extracted from DENV-2-infected cells and reverse-transcribed into cDNAs. The expression levels of upregulated transcripts (**A**) and downregulated transcripts (**B**) were validated using qRT-PCR, using human GAPDH as the reference gene. Data were presented as mean ± SEM from three independent experiments. (**C**) HDFs were treated with UV-inactivated DENV-2 HDF supernatant for 24 h, followed by ZIKV infection at an MOI of 5 for 24 h. ZIKV infectivity was quantified using flow cytometry. (**D-F**) Production levels of IFN-β, IFN-γ, and CXCL10 in DENV-2-infected HDF supernatant were quantitated using ELISA. Data were presented as mean ± SEM from three independent experiments and analyzed using Student’s *t*-test (*p* < 0.05)

To validate the accuracy of transcriptomic profile, the up- or down-regulation of transcripts were monitored using qRT-PCR. The upregulation of 14 transcripts and 6 downregulated transcripts were quantitated. As shown in Fig. 6A and B, the expression levels of key genes involved in interferon responses (*IFNB1*, *RSAD2*, and *STAT1*), ISG-mediated antiviral pathways (*OAS1*, *OAS2*, *MX2*, *ISG15*, and *IFIT2*), cytokine and chemokine-related inflammatory responses (*TNF*, *IL1B*, *CXCL10*, and *CXCL8*), and immune-related genes (*ACOD1* and *EGR4*) were upregulated during DENV-2 infection (Fig. 6A and Additional file 7). Moreover, genes related to cell cycle processes (*CCNB2*, *CCNA2*, *BUB1B*, *TOP2A*, and *CENPF*) and clathrin-mediated endocytosis (*CHRM2*) were downregulated (Fig. 6B and Additional file 7). These findings confirmed the accuracy of transcriptomic profile.

Furthermore, transcriptome results revealed that DENV-2-infected HDFs activated gene expression related to innate immune response, suggesting that prior DENV-2 infection could induce an antiviral state within the infected cells as well as the uninfected neighboring cells. To validate this phenomenon, the biological activity of that DENV-2-infected HDFs supernatant was performed. HDFs cultures were treated with UV-inactivated DENV-2 HDF supernatant for 24 h and then infected with ZIKV. The results showed that treated HDFs significantly reduced ZIKV infection compared to mock-treated cells (Fig. 6C). To identify secreted immune mediators in the DENV-2-infected HDFs supernatant that potentially induced an antiviral state in the neighboring cells, we quantified the type I IFN, particularly IFN-β, which plays a central role in antiviral responses in human skin cells. Additionally, type II IFN (IFN-γ) and chemokine CXCL10, which were significantly upregulated in our transcriptome results and might be antiviral cytokine/chemokine were also quantified using ELISA. DENV-2 infection strongly induced the production of IFN-β, IFN-γ, and CXCL10 compared to the mock-infected controls (Fig. 6D-F).

## Discussion

The mosquito-borne arboviruses such as DENV, ZIKV, and CHIKV are expanding their territory and hence are of public health concern. These viruses are heavily dependent on the same mosquito vector. Indeed, coinfection of these viruses in the mosquito vectors and in human host have been reported in areas where these viruses co-circulate [1–4]. Studies on coinfection between DENV and ZIKV in mosquito host strongly support the superinfection exclusion phenomenon [13], in which the prior viral infection significantly reduced production of the subsequent-infected virus [17]. How this coinfection and interplay between the two viruses within a mosquito host contribute to disease outbreaks remains unclear.

Thailand is the hyperendemic area of DENV in which the four serotypes of DENV co-circulate year-round, while epidemics of ZIKV and CHIKV sporadically occur every 5–10 years. With these situations, patients co-infected with DENV and ZIKV as well as DENV and CHIKV have been reported [3, 18]. With the limiting numbers of co-infected patients, it is suggested that co-infection between DENV/ZIKV or DENV/CHIKV did not accelerate disease severity [3]. This stimulated us to investigate the interplay between DENV and ZIKV at their entry site in the human host. We developed the in vitro simultaneous- and sequential-coinfection of DENV-2 and ZIKV using the primary HDF cells, the first line of skin cells that encounter with these viruses during mosquito feeding [19, 20]. Unexpectedly, during simultaneous coinfection between DENV-2 and ZIKV, the suppressive effect was observed on ZIKV RNA accumulation but no effect was seen on DENV-2 RNA levels. A similar effect was found for sequential coinfection in which DENV-2 dominantly replicated comparing to that of ZIKV. The ZIKV infection exclusion may result from mechanisms beyond RNA synthesis, such as limited viral entry, reduced RNA stability, hindered translation of viral proteins, or interference with the formation of viral replication organelles. Nonetheless, the precise stage at which ZIKV RNA production is diminished remains to be elucidated.

The potential mechanisms for viral interference during coinfection have been reported. For example, JEV NS2B protein inhibited superinfection of ZIKV by competing for cellular resources [21]. The other mechanisms have been elucidated including activation of antiviral defense [8, 9, 12], blocking cell surface receptors [22], virus-induced cellular state resistance to subsequent infection, and competition for host factors supporting replication [17, 23]. As shown in our results, DENV-2 exerted interferent effect on ZIKV during sequential coinfection stronger than that of simultaneous coinfection. Therefore, we addressed the potential pathways involving in sequential coinfection exclusion through monitoring the transcriptomic changes in DENV-2-infected HDFs at both 16 and 24 hpi. The altered transcripts were used to hypothesize potential interference mechanisms induced by DENV-2 that might contribute to the suppression of ZIKV during sequential coinfection. We revealed, here, that 82.2% of the altered transcripts were upregulated genes enriched in innate immune response and nucleosome signaling pathways, while 17.8% of the downregulated genes were associated with cell cycle and viral entry pathways. With these results, three mechanisms of the replication exclusion were hypothesized.

The first mechanism entailed the strong activation of the innate anti-viral responses. This potential mechanism was supported by previous findings on the coinfection of respiratory viruses, where type I and III IFNs, triggered by primary influenza virus or RSV infection, inhibit SARS-CoV-2 replication [8, 9, 12]. Our transcriptome profile revealed significant activation of the IFN signaling cascade (upregulation of *OAS1*, *OAS2*, *MX2*, *ISG15*, and *IFIT2*), driven by highly expressed pattern-recognition receptor (PRR) signaling pathways that induce the expression and secretion of IFNs. Notably, our findings revealed the dominant triggering of IFN-β and IFN-γ, which are key components of type I and II IFNs, respectively. IFN-β is the early antiviral response in skin cells, while IFN-γ generates additional protective antiviral activities through independent receptor from IFN-β [24]. It is generally known that IFN production is quite delayed during flaviviruses infection [25]. For instance, induction of type I IFN was observed in DENV-2-infected dendritic cells at 36 h post-infection [26]. However, this may depend on cell type as both type I and II IFN and other anti-viral innate mediators were upregulated in DENV-2-infected HDF cells as early as 16 h post-infection (Fig. 6D-E). In addition, we showed that UV-inactivated supernatant of DENV-2-infected HDFs exerted resistance to subsequent ZIKV infection (Fig. 6C). Previous studies have also demonstrated that pre-treatment with IFN significantly inhibits ZIKV infection [27, 28]. In addition, the transcriptomic profile also revealed upregulation of transcripts involving in nucleosome signaling pathway which can regulate innate immunity by promoting the expression of antiviral genes such as cytokines and interferons [29–31]. Thus, our transcriptomic data suggested that DENV-2 infection may drive HDF cells into the anti-viral state via activation of anti-viral innate responses. This may be the major mechanism that DENV interferes with ZIKV RNA accumulation during sequential- but not simultaneous-coinfection. This conclusion is supported by our transcriptomic results which showed that 77% of total altered transcripts were innate anti-viral related genes.

The second hypothetical interference mechanism is blocking the entry process of ZIKV superinfection. Upon primarily infected by DENV-2, downregulation of genes related to the ephrin receptor and clathrin-mediated endocytosis were observed. These molecules are known to involve in the entry process of various groups of viruses [32–36]. Significantly, we showed that DENV-2 infected HDF cells reduced not only clathrin-mediated endocytosis but also the expression of *TYRO3*, a receptor for ZIKV [20]. Our results suggested that DENV-2 may inhibit entry of ZIKV during coinfection. This mechanism has been proposed in superinfection exclusion of various viruses [22, 37].

The third hypothetical mechanism may be cell cycle disruption by DENV-2 resulting in a cellular stage that cannot support subsequent ZIKV replication. DENV-2 and ZIKV exploit host factors at distinct stages of the cell cycle for their replication. DENV-2 infection disrupts the cell cycle by inducing arrest at the G1/S checkpoint in HuH-7 cells and BHK-21 cells [38, 39], whereas ZIKV causes G2/M cell cycle arrest [40–43]. Choksupmanee et al. have shown that DENV RNA tightly binds to DDX6, a DEAD-box RNA helicase, resulting in G1 phase arrest and suppression of S phase entry [38], creating a favorable intracellular environment for DENV replication. In contrast, ZIKV requires host factors enriched during G2/M phase for its growth. The envelop protein and nonstructural protein 5 of ZIKV were shown to inhibit Cyclin B1/CDK1 activity and regulates the mitotic spindle activity, respectively [41, 42], resulting in G1/M phase arrest. In our present study, genes involving in cell cycle progression were shown to be suppressed in DENV-2-infected HDF cells. This supported DENV-2 replication but in turn created an environment unfavorable for ZIKV replication. Thus, DENV-2-induced cell cycle modulation could be another mechanism that may participate in suppression of ZIKV superinfection.

In simultaneous coinfection, we demonstrated that DENV-2 RNA production was unaffected, whereas ZIKV RNA accumulation in culture supernatant was markedly suppressed. Unfortunately, the transcriptomic data obtained at 16 and 24 hpi were too late to capture the possible mechanism that mediated ZIKV suppression. However, the mechanisms underlying negative interaction during coinfection have been reported by various studies. Among these, the immune responses were shown to have the central role in establishing the negative interaction. To observe such effect, however, it is crucial to allow sufficient time between the first and second viral infection. Therefore, negative interaction between DENV-2 and ZIKV during simultaneous coinfection in our experimental setting was unlikely to be mediated through the immune responses. Nevertheless, role of intrinsic cellular defenses, such as RNAi defense system, cannot be excluded [44, 45]. Beside the immune responses, other mechanisms such as competition for limited cellular resources has been investigated in several arbovirus coinfection. For example, coinfection between DENV-2 and ZIKV in mosquito cells revealed the enhancement of DENV replication whereas ZIKV was suppressed. The mechanism underlying this negative interaction is the ability of DENV genome to competitively engage the ZIKV replicative complex [14]. In our investigated model, DENV-2 infection at the MOI of 5 established genome replication earlier than that of ZIKV infection at the MOI of 10 (Additional file 2). With the rapid and more efficient replication, DENV may shaping a cellular environment that favor its own growth and outcompeting ZIKV for cellular resources. How does DENV-2 outcompete ZIKV in simultaneous coinfection in HDF cells required further elucidation.

## Conclusions

Our study demonstrated that primary DENV-2 infection in HDF cells induced exclusion of superinfection, thereby ZIKV RNA accumulation was suppressed. Three potential mechanisms underlying this phenomenon are proposed. The first proposed mechanism is activation of the innate antiviral response to block further infection. The second mechanism is the downregulation of ZIKV receptors and the endocytic pathway, thereby preventing viral entry. The last hypothetical mechanism is primary DENV-2-infection induces cell cycle arrest, which favors its own replication while limiting resources for ZIKV replication. The first mechanism can inhibit subsequent infection in the infected- and the non-infected bystander cells while the other two mechanisms may act specifically on protecting the DENV-2-infected cells from ZIKV superinfection. Further investigations are required to explore these hypothesized pathways in greater detail and clarify their contributions to viral restriction. The limitation of our study is the use of bulk transcriptomic method. One of disadvantages of this technique is unable to indicate whether expression changes occurred in the infected cells or bystander cells or both. However, the bulk transcriptomics effectively captured the major events that may occur during coinfection in skin cells. Further study using single-cell transcriptomic analysis to clarify differential gene expression between infected and bystander cells may be performed.

## Electronic supplementary material

Below is the link to the electronic supplementary material.

Supplementary Material 1: Additional file 1. List of gene-specific primers used in qRT-PCR.

Supplementary Material 2: Additional file 2. The viral replication kinetics of DENV-2 or ZIKV monoinfection in HDFs. HDFs were infected with DENV-2 at MOI of 5 or ZIKV at MOI of 10. The culture supernatant was harvested at 6, 12, 24, and 48 hpi. Viral RNA copies were quantified using qRT-PCR.

Supplementary Material 3: Additional file 3. Infectivity of DENV-2 infection in HDFs at 16 and 24 hpi. HDFs cultures were infected with DENV-2 and infectivity were observed at 16 and 24 hpi using flow cytometry (A). The percentage of infectivity was shown in (B). Data were presented as mean ± SEM from three independent experiments.

Supplementary Material 4: Additional file 4. Data landscape of transcriptomic changes in HDFs response to DENV-2 infection. Quantile-transformed read counts of samples at 16 and 24 hpi.

Supplementary Material 5: Additional file 5. Functional enrichment analysis of Gene Ontology (GO) terms enriched with total DEGs at 16 hpi. Top 10 enriched pathways at 16 hpi based on GO for biological process (A), cellular component (B), and molecular function (C). Color dots indicate significant enrichment (p-value ≤ 0.05). Dot size indicates the gene counts.

Supplementary Material 6: Additional file 6. Clusters of upregulated DEGs at 16 hpi potentially implicated in viral interference. The associations among upregulated DEGs, PPI networks, were generated using the STRING database and visualized in Cytoscape. Module 1 (A), module 2 (B), and module 3 (C) of the upregulated genes in DENV-2-infected HDFs at 16 hpi were identified as highly interconnected clusters using MCODE.

Supplementary Material 7: Additional file 7. The expression levels of genes at 16 and 24 hpi based on RNA-seq data.

## Data Availability

No datasets were generated or analysed during the current study.

## Abbreviations

DENV: Dengue virus
ZIKV: Zika virus
HDFs: Human dermal fibroblasts
RSV: Respiratory syncytial virus
CHIKV: Chikungunya virus
MOI: Multiplicity of infection
hpi: Hours post infection
qRT-PCR: Quantitative RT-PCR
MFI: Mean fluorescence intensity
iDEP: Integrated differential expression and pathway analysis
DEGs: Differentially expressed genes
GO: Gene Ontology
PPI: Protein-protein interaction
MCODE: Molecular Complex Detection
PRRs: Pattern-recognition receptors
ISGs: Interferon-stimulated genes (ISGs)
