# Supplementary material for: Transcriptomic analysis of DENV-2-infected human dermal fibroblasts identified potential mechanisms that suppressed ZIKV replication during sequential coinfection
Source: Virol J. 2025 May 22;22:154. doi: 10.1186/s12985-025-02769-9 (PMC12096689; doi:10.1186/s12985-025-02769-9)
Supplement: Supplementary file 1 — Supplementary Material 1: Additional file 1. List of gene-specific primers used in qRT-PCR. [file 12985_2025_2769_MOESM1_ESM.pdf]

Additional file 1. List of gene-specific primers used in qRT-PCR.

Gene	Primer sequence (5' - 3')	Source
DENV-2	F' CAATATGCTGAAACGCGAGAG<br>R' GTGGGATTGTTAGGAAACGAA	Limthongkul J. et al., 2023
ZIKV	F' AAGTACACATACCAAAACAAAGTGGT<br>R' CCACTAGGTTGGTAAATGTGT	Garcia G. et al., 2023 and NCBI primer BLAST
Beta-actin	F' CCTGGCACCCAGCACAAT<br>R' GGGCCGGACTCGTCATAC	Modhiran N. et al., 2010
IFNβ1	F' GCGACACTGTTCGTGTTGTC<br>R' AGCCTCCCATTCAATTGCCA	Han N. et al., 2021
RSAD2	F' CCCCAACCAGCGTCAACTAT<br>R' TGATCTTCTCCATACCAGCTTCC	Rand U. et al., 2021
STAT1	F' TCTGTGTCTGAAGTTCACCCTT<br>R' GAAAACTGTCGCCAGAGAAGA	NCBI primer BLAST
OAS1	F' TCCTGGATTCTGCTGGCTGAAAG<br>R' CCTGGAGTGTGCTGGGTCTAT	NCBI primer BLAST
OAS2	F' CTTTGACATTGCTGAAGGCGT<br>R' CCAAGATTACTGGCCTCGCT	NCBI primer BLAST
MX2	F' TGAACGTGCAGCGAGCTT<br>R' AGAAAATTGACTTCTCCTCCGGT	Sugai A. et al., 2017 and NCBI primer BLAST
ISG15	F' ACAGCCATGGGCTGGGA<br>R' GCCAGACGCTGCTGGAA	Wick C. et al., 2023 and NCBI primer BLAST
IFIT2	F' ACTGCAACCATGAGTGAGAACA<br>R' CGATTCTGAAACTCAGTCCGGT	NCBI primer BLAST
TNF	F' GCCCATGTTGTAGCAAACCCT<br>R' GGACCTGGGAGTAGATGAGGT	NCBI primer BLAST
IL-1β	F' TGAAATGATGGCTTATTACAGTGGC<br>R' CCTTGCTGTAGTGGTGGTCG	Zhang M. et al., 2023 and NCBI primer BLAST
CXCL10	F' CCACGTGTTGAGATCATTGCT<br>R' TGCATCGATTTTGCTCCCCT	Schiantarelli J. et al., 2025
CXCL8	F' TGGAGAAGTTTTTGAAGAGGGCT<br>R' CAACAGACCCACACAATACATGA	Fang X. et al., 2024
ACOD1	F' CAACGAAATGATGCTCAAGTCTATC<br>R' CTTCCGTAGTGGTTCCCAGG	NCBI primer BLAST
EGR4	F' TCCACCTTAGCGAGTTTTCCG<br>R' TCAAGAAGTCGCCTGCTCCAG	NCBI primer BLAST

CCNB2	F' GAAAAGTTGGCTCCAAAGGGTC<br>R' TCACTGCAGAGCTGAGGGTT	NCBI primer BLAST
CCNA2	F' AAGACGAGACGGGTTGCAC<br>R' AGCTGGCTTCTTCTGAGCTT	NCBI primer BLAST
BUB1B	F' CCTGGCTAACTGTTCTTCTCCC<br>R' CACAATTCACCATCTTTTAGCTCAG	NCBI primer BLAST
TOP2A	F' GTGTCACCATTGCAGCCTGT<br>R' ACCAATGTAGGTGTCTGGGC	Chen X. et al., 2020 and NCBI primer BLAST
CENPF	F' CCGTCCCCGAGAGCAAGTTTA<br>R' TCAAGCTGTCCTTCAAGCTCT	NCBI primer BLAST
CHRM2	F' CGAATAGGTGTTGCCTCCCAA<br>R' TCTCTTTCTCCCTTGATCTGGC	NCBI primer BLAST
Human GAPDH	F' GTGGACCTGACCTGCCGTCT<br>R' GGAGGAGTGGGTGTCGCTGT	NCBI primer BLAST
