# Supplementary figures and images for: Transcriptomic analysis of DENV-2-infected human dermal fibroblasts identified potential mechanisms that suppressed ZIKV replication during sequential coinfection
Source: Virol J. 2025 May 22;22:154. doi: 10.1186/s12985-025-02769-9 (PMC12096689; doi:10.1186/s12985-025-02769-9)

## Viral replication

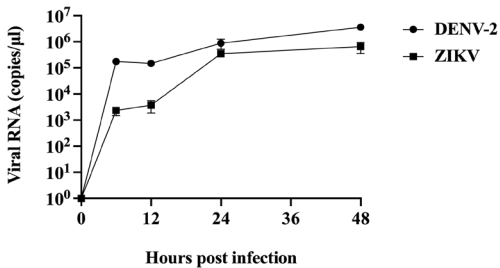

Supplement: Supplementary file 2 — Supplementary Material 2: Additional file 2. The viral replication kinetics of DENV-2 or ZIKV monoinfection in HDFs. HDFs were infected with DENV-2 at MOI of 5 or ZIKV at MOI of 10. The culture supernatant was harvested at 6, 12, 24, and 48 hpi. Viral RNA copies were quantified using qRT-PCR. [file 12985_2025_2769_MOESM2_ESM.pdf]

**(A)**

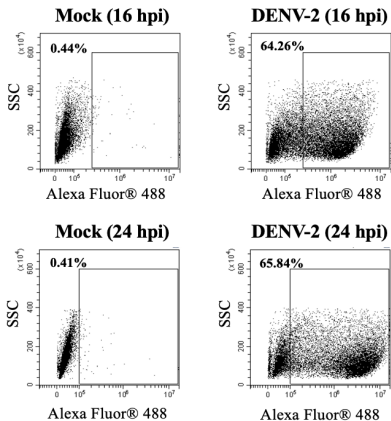

**(B)**

**DENV-2 infection in HDFs**

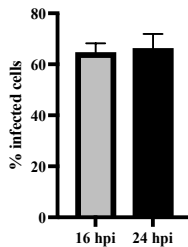

Supplement: Supplementary file 3 — Supplementary Material 3: Additional file 3. Infectivity of DENV-2 infection in HDFs at 16 and 24 hpi. HDFs cultures were infected with DENV-2 and infectivity were observed at 16 and 24 hpi using flow cytometry (A). The percentage of infectivity was shown in (B). Data were presented as mean ± SEM from three independent experiments. [file 12985_2025_2769_MOESM3_ESM.pdf]

Distribution of transformed data

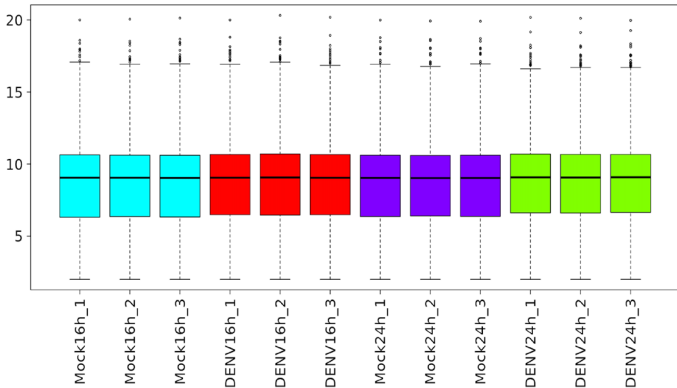

Supplement: Supplementary file 4 — Supplementary Material 4: Additional file 4. Data landscape of transcriptomic changes in HDFs response to DENV-2 infection. Quantile-transformed read counts of samples at 16 and 24 hpi. [file 12985_2025_2769_MOESM4_ESM.pdf]

**(A)**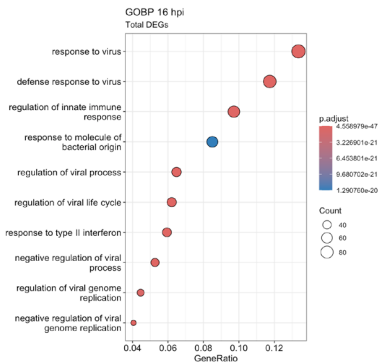**(B)**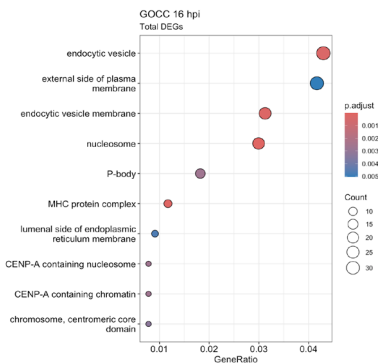**(C)**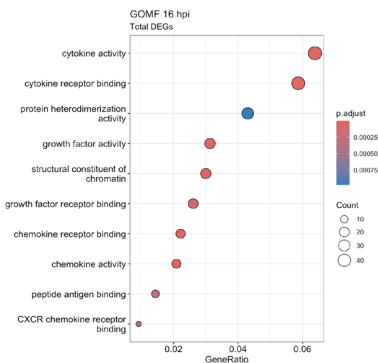

Supplement: Supplementary file 5 — Supplementary Material 5: Additional file 5. Functional enrichment analysis of Gene Ontology (GO) terms enriched with total DEGs at 16 hpi. Top 10 enriched pathways at 16 hpi based on GO for biological process (A), cellular component (B), and molecular function (C). Color dots indicate significant enrichment (p-value ≤ 0.05). Dot size indicates the gene counts. [file 12985_2025_2769_MOESM5_ESM.pdf]

(A) Module 1

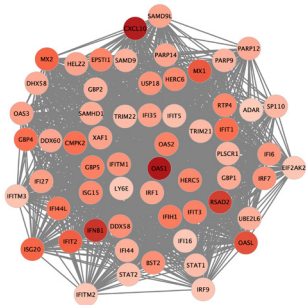

(B) Module 2

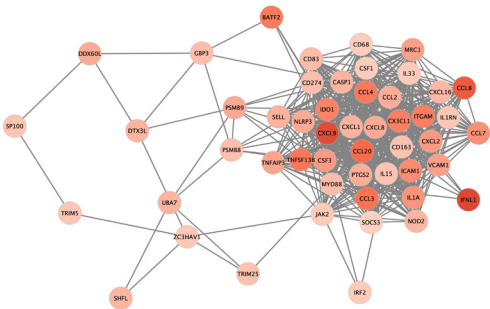

(C) Module 3

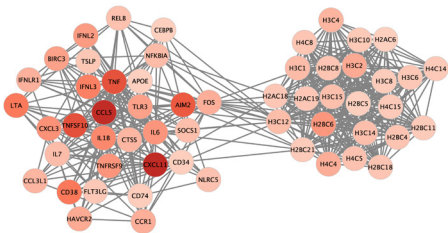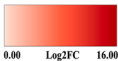

Supplement: Supplementary file 6 — Supplementary Material 6: Additional file 6. Clusters of upregulated DEGs at 16 hpi potentially implicated in viral interference. The associations among upregulated DEGs, PPI networks, were generated using the STRING database and visualized in Cytoscape. Module 1 (A), module 2 (B), and module 3 (C) of the upregulated genes in DENV-2-infected HDFs at 16 hpi were identified as highly interconnected clusters using MCODE. [file 12985_2025_2769_MOESM6_ESM.pdf]
