# Supplementary material for: Transcriptomic analysis of DENV-2-infected human dermal fibroblasts identified potential mechanisms that suppressed ZIKV replication during sequential coinfection
Source: Virol J. 2025 May 22;22:154. doi: 10.1186/s12985-025-02769-9 (PMC12096689; doi:10.1186/s12985-025-02769-9)
Supplement: Supplementary file 7 — Supplementary Material 7: Additional file 7. The expression levels of genes at 16 and 24 hpi based on RNA-seq data. [file 12985_2025_2769_MOESM7_ESM.pdf]

Additional file 7. The expression levels of genes at 16 and 24 hpi based on RNA-seq data.

Regulation	Gene	Functional enrichment	log2FC (16 h)	log2FC (24 h)
Up	*IFNB*	Interferon pathway	12.65	14.82
	RSAD2		11.81	9.96
	STAT1		2.36	2.47
	OAS1	ISGs-mediated antiviral pathway	15.20	13.38
	OAS2		6.31	5.87
	MX2		8.41	7.53
	ISG15		5.67	5.95
	IFIT2		8.86	8.30
	TNF	Cytokine activity	10.03	12.37
	IL1B		5.88	6.67
	CXCL10	Chemokine activity	14.71	15.94
	CXCL8		3.57	8.32
	ACOD1	Immune response	9.30	11.91
	EGR4		8.89	11.40
Down	*CCNB2*	Cell cycle process	-0.41	-1.15
	CCNA2		-0.66	-1.12
	TOP2A		-0.61	-1.00
	BUB1B		-0.57	-1.11
	CENPF		-0.51	-1.09
	CHRM2	Clathrin-mediated endocytosis	-0.87	-2.71
